# Constitutive *RB1 *mutation in a child conceived by *in vitro *fertilization: implications for genetic counseling

**DOI:** 10.1186/1471-2350-10-75

**Published:** 2009-07-29

**Authors:** Raquel H Barbosa, Fernando R Vargas, Evandro Lucena, Cibele R Bonvicino, Héctor N Seuánez

**Affiliations:** 1 Genetics Division, Instituto Nacional de Câncer, Rua André Cavalcanti, 37, 4th floor, 20231-050 Rio de Janeiro, RJ, Brazil; 2 Post-graduate Program in Oncology, Instituto Nacional de Câncer, 20231-050 Rio de Janeiro, Brazil; 3 Department of Genetics, Universidade Federal do Estado do Rio de Janeiro, 22290-240 Rio de Janeiro, Brazil; 4 Paediatric Service, Instituto Nacional de Câncer, 20230-030 Rio de Janeiro, Brazil; 5 Department of Genetics, Universidade Federal do Rio de Janeiro, 21941-901 Rio de Janeiro, Brazil

## Abstract

**Background:**

The purpose of this study was to identify mutations associated with bilateral retinoblastoma in a quadruplet conceived by in vitro fertilization, and to trace the parental origin of mutations in the four quadruplets and their father.

**Methods:**

Mutational screening was carried out by sequencing. Genotyping was carried out for determining quadruplet zygosity.

**Results:**

The proband was a carrier of a novel *RB1* constitutive mutation (g.2056C>G) which was not detected in her father or her unaffected sisters, and of two other mutations (g.39606 C>T and g.174351T>A) also present in two monozygotic sisters. The novel mutation probably occurred de novo while the others were of likely maternal origin. The novel mutation, affecting the Kozak consensus at the 5'UTR of *RB1* and g.174351T>A were likely associated to retinoblastoma in the proband.

**Conclusion:**

Molecular diagnosis of retinoblastoma requires genotypic data of the family for determining hereditary transmission. In the case of children generated by IVF with oocytes from an anonymous donor which had been stored in a cell repository, this might not be successfully accomplished, making precise diagnosis impracticable for genetic counseling.

## Background

Retinoblastoma (RB; MIM #180200) is the most common intraocular pediatric tumor, with an incidence of 1/15,000–25,000 live births. It results from mutational inactivation of both alleles of the tumor suppressor *RB1 *gene that encodes a nuclear phosphoprotein (pRB) involved in the control cell cycle [1-3]. In the hereditary form, accounting for 40% of RB patients, an *RB1 *germline mutation is transmitted as an autosomal dominant trait with high penetrance (90%), resulting in a 45% risk of occurrence in the offspring, with increased risk for secondary malignancies. In the sporadic form, mutational inactivation of both *RB1 *alleles arises from somatic events in retinal cells, without germline alterations [4].

Genetic alterations frequently resulting from *RB1 *inactivation involve chromosome rearrangements affecting the 13q14 region (deletion, translocation), nucleotide changes (substitutions, deletions, insertions and duplications), exonic deletions (single or multiple); loss of heterozygosity [5], or CpG island hypermethylation of the *RB1 *promoter region [6]. Epigenetic mechanisms playing a role in some RB patients include differential methylation of chromosome 13q around *RB1 *[7] and preferential loss of maternal alleles in sporadic cases, suggesting a latent imprinting [8].

Identification of *RB1 *mutations in hereditary RB provides accurate risk prediction and valuable genetic counseling for patients and their families. Furthermore, the nature of a mutation can determine genetic penetrance, disease presentation and prognosis [9].

Recent investigations suggest an apparent association between *in vitro *fertilization (IVF) and genetic syndromes, including retinoblastoma [8]. It has been postulated that embryos in culture may acquire epigenetic defects as a result of abnormal environmental conditions which may lead to aberrant phenotypes. These studies illustrated a complex problem involving legal and ethical aspects and privacy protection in the field of assisted reproductive technologies (ART), with potential implications in the genetic counseling of retinoblastoma.

We herein describe a novel constitutive mutation at the 5' UTR of *RB1 *in a patient with bilateral retinoblastoma, who also carried two other constitutive mutations also present in two of her sisters conceived by IVF.

## Methods

### Patients and samples

We studied a family of female quadruplets (A, B, C and D) conceived by IVF of oocytes from one anonymous donor with sperm of their father. One quadruplet presented bilateral retinoblastoma diagnosed by current ophthalmological and histopathological criteria at one month of age. The proband (A) was referred to the Genetic Counseling Program of the Instituto Nacional de Cancer (Rio de Janeiro, Brazil) and subsequently selected for mutational screening, together with her three sisters and their father. All quadruplets have been followed by periodical ophthalmological examinations to present.

Blood samples were obtained from the quadruplets and their father. Samples and information on the family were obtained with an informed consent. Tumor samples were not available for analysis. With respect to privacy and anonymity required for IVF, blood samples could not be obtained from the female donor. This study was approved by local Ethics Committee and followed the tenents and guidelines of the Declaration of Helsinki.

### Mutation screening

*RB1 *exons 2–27 were PCR-amplified in individual reactions containing 100 ng DNA, 0.4 pm of each primer [forward and reverse previously reported [5]], 0.15 mM of each dNTP, 3 mM MgCl_2_, 1× PCR buffer (Promega Corporation, Madison, Wisconsin, USA) and 1 U of *Taq *DNA polymerase (Promega Corporation) in 50 μl reactions. PCR was performed with 40 cycles at 94°C (1 min), 54°C (40 sec) and 72°C (35 sec) in a programmable thermocycler (PT-100; MJ Research, Inc., Waltham, Massachussets, USA). Reaction mixes were prepared as previously described [5], with *Taq *DNA Polymerase, Recombinant (Invitrogen, Carlsbad, California, USA). PCR conditions were 40 cycles at 94°C (1 min), 49°C (40 sec) and 72°C (35 sec), in a programmable thermocycler (PT-100; MJ Research, Inc.).

### *RB1 *promoter region and exon 1 analysis

Forward 5'-GGTTTTTCTCAGGGGACGTT-3' and reverse 5'-AACCCAGAATCCTGTCACCA-3' primers were used to amplify the *RB1 *promoter and exon 1 from genomic DNA. Reaction mixes were prepared containing 100 ng DNA, 0.4 pm of each primer (forward and reverse), 5% DMSO, 0.15 mM of each dNTP, 3 mM MgCl_2_, 1× PCR buffer (Promega Corporation, Madison, Wisconsin, USA) and 1 U of Platinum *Taq *DNA polymerase (Promega Corporation) in 50 μl reactions. PCR was performed with 40 cycles at 94°C (1 min), 50°C (40 sec) and 72°C (45 sec) in a programmable thermocycler (PT-100; MJ Research, Inc., Waltham, Massachussets, USA).

RNA was extracted from blood cells of the proband isolated with Ficoll-Paque PLUS^® ^(GE Healthcare UK, Little Chalfont, UK) using TRIZOL^® ^(Invitrogen Corporation, California, USA) following the specifications of the manufacturer and cDNA was synthesized with SuperScript First-Strand Synthesis System for RT-PCR (Invitrogen Corporation, California, USA).

A cDNA region, including part of the 5' UTR of *RB1*, was amplified using the forward primer 5'-CTCCCCGGCGCTCCTCCACAGC-3' annealing downstream of transcription start sites -176 and -128 [10] and the reverse primer 5'-AGAACACCACGAAAAAGTAA-3' annealing in exon 6. Reaction mixes contained 100 ng cDNA, 0.4 pm of each primer, 5% DMSO, 0.15 mM of each dNTP, 3 mM MgCl2, 1× PCR buffer (Promega Corporation, Madison, Wisconsin, USA) and 1 U of Platinum Taq DNA polymerase (Promega Corporation) in 50 μl reactions. PCR was performed with 50 cycles at 94°C (1 min), 55°C (40 sec) and 72°C (1 min) in a programmable thermocycler (PT-100; MJ Research, Inc., Waltham, Massachussets, USA).

Amplified products were electrophoresed in 2% agarose gels with ethidium bromide. Direct sequencing was performed with an ABI PRISM 377 automatic DNA sequencer (Applied Biosystems, Foster City, California, USA). Sequences were analyzed using Sequencher TM Demo Version 4.1.4 software (Gene Codes Corporation 2005 – 775 Technology Drive, Ann Arbor, Michigan, USA) and compared to a reference sequence (Genbank accession L11910.1). Constitutive alterations identified in this study were compared with those published in *RB1 *gene mutation database-Retinoblastoma Genetics website (available at ).

### Diagnosis of zygosity

Genotyping was carried out with PowerPlexTM16 System (Promega Corporation), following the recommendations of the manufacturer, for identifying quadruplet zygosity. This assay comprised multiplex PCR amplifications of 15 STR loci, including D13S317 (a microsatellite marker located at 13q22-q31). An additional microsatellite flankling *RB1 *(D13S284 located at 13q14.3) was also used for genotyping.

## Results

**Figure 1:**
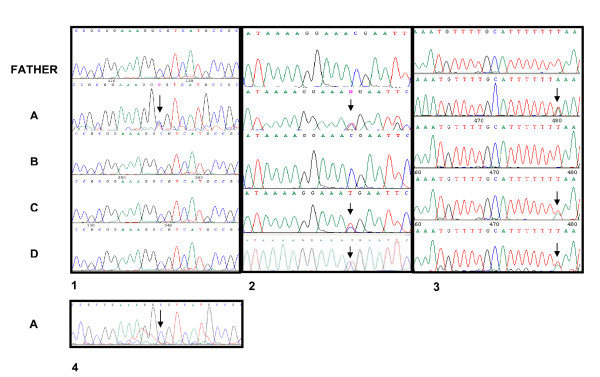
**1. Partial sequence of 5' UTR of *RB1 *in genomic DNA showing a C>G mutation (indicated by arrow) in position -4 with respect to the initial translation ATG codon in proband (A)**. This alteration was not detected in the father or in other quadruplets (B, C and D). **2**. Partial sequence of intron 3 in genomic DNA showing a C>T transition (indicated by arrow) in proband A and in two monozygotic sisters (C and D). This transition was absent in the father and B. **3**. Partial sequence of intron 25 in genomic DNA showing a T>A transversion (indicated by arrow) in the proband A and two monozygotic sisters C and D. This transversion was absent in the father and quadruplet B. **4**. Partial sequence of 5' UTR of *RB1 *in cDNA of proband's leukocytes showing only the g.2056C allele.

Fundoscopic analysis identified a bilateral retinoblastoma in one child (A) while the three other quadruplets and their father showed a normal fundoscopy. Mutational screening in the proband detected three constitutive alterations, one consisting of a transversion in intron 25 (g.174351T>A), previously reported [11,12], and a transition in intron 3 (g.39606 C>T), previously described [7]. Both mutations were also present in quadruplets C and D but were absent in B and their father (Figure 1). A third mutation consisted of a novel transversion at the 5' UTR of the *RB1 *promoter, in position – 4 (g.2056C>G) with respect to the initial ATG codon. This mutation was not detected in her father or the three other quadruplets. Analysis of the proband's cDNA showed a clear electropherogram showing only the g.2056C allele (Figure 1).

Genotyping showed that the quadruplets had originated from three trizygotic embryos, one corresponding to A, a second one to B, and a third one to the monozygotic twins C and D. One 13q marker (D13S317) included in the PowerPlex 16 system showed two paternal alleles (11 pb and 12 pb), and one maternal allele (9 pb) that was present in all quadruplets (Table 1). These results were confirmed by D13S284, with two paternal alleles (213 pb and 215 pb) and two maternal alleles (205 pb and 213 pb; see Table 2).

**Table 1:** Genotyping of four quadruplets and father.

**Locus**	**Father**	***Proband *(A)**	**Sister (B)**	**Sister (C)**	**Sister (D)**	**Deduced maternal alleles**
D3S1358	14–18	17–18	14–17	14–17	14–17	17–?

TH01	7–8	7–9	7–9.3	8–9.3	8–9.3	9–9.3

D21S11	28–29	28–31	29–31	28–31	28–31	31 – ?

D18S51	14–15	14–15	14–15	14–15	14–15	14? – or 15?

PENTA E	13–15	13–15	5–13	13–15	13–15	5 – ?

D5S818	12–12	12–13	12–12	12–13	12–13	12–13

D13S317	11–12	9–11	9–12	9–12	9–12	9 – ?

D7S820	11–12	11–12	11–11	11–12	11–12	11–?

D16S539	8–9	8–12	9–12	9–9	9–9	9–12

CSF1PO	10–12	10–11	10–12	10–10	10–10	11–10

PENTA D	2.2–10	10–13	10–13	2.2–13	2.2–13	13 – ?

VWA	16–16	16–16	13–16	13–16	13–16	13–16

D8S1179	11–13	11–13	9–11	13–13	13–13	9–13

TPOX	8–11	8–11	11–11	8–11	8–11	11 – ?

FGA	21–25	21–21	21–21	21–21	21–21	21–?

AMEL	X-Y	X-X	X-X	X-X	X-X	

? = unknown allele

Analysis of 16 molecular markers used for genotyping quadruplets and their father. Numbers represent alleles identified with PowerPlex 16 System^®^. Maternal alleles were deduced. The Amelogenin marker (AMEL) was used as an X chromosome marker. Genotyping allowed for the diagnosis of zygosity in the quadruplets.

**Table 2:** Genotyping of quadruplets and father with respect to mutations and 13q markers.

Mutation/marker	Alleles				
	
	Father	A (proband)	B	C	D
g.2056C>G (in – 4 position in 5'UTR)	C/C	G/**C**	C/**C**	C/**C**	C/**C**

g.39606C>T (in intron 3)	C/C	T/**C**	C/**C**	T/**C**	T/**C**

g.174351T>A (in intron 25)	T/T	A/**T**	T/**T**	A/**T**	A/**T**

D13S284	213/215	213/**213**	205/**215**	213/**215**	213/**215**

D13S317	11/12	9/**11**	9/**12**	9/**12**	9/**12**

D13S284 showed two paternal alleles (213 pb and 215 pb) and two maternal alleles (205 pb and 213 pb). The maternal allele 213 pb is present with g.2056G in the proband (A), with g.39606T and with g.174351A in quadruplets A, C and D. Paternal alleles in the quadruplets are indicated in bold characters.

## Discussion

Several clinical reports have suggested an association between assisted reproduction technologies (ART) and increased risk of genetic disorders including retinoblastoma, in children conceived with the use of ART. These might be a consequence of accumulation of epigenetic alterations during embryo culture and/or alteration of developmental timing. In some cases of retinoblastoma, *RB1 *was epigenetically silenced by hypermethylation at the promoter region, resulting in reduced *RB1 *expression. Since tumor material was not available, peripheral assessment of methylation of the RB1 promoter in leukocytes would not have been informative because the dynamics of DNA methylation in primary tumor tissues are clearly different from normal cells, and not confined to specific CpG sites [13].

### *RB1 *patterns and haplotypes

Genotypic analysis of 13q was carried out with three intragenic *RB1 a*lleles and two microsatellites closely linked to *RB1*. Presumably, transmission of intragenic alleles occurred without recombination, corresponding to parental *RB1 *patterns [C-C-T] and [C-T-A]. Conversely, the possibility of recombination might have occurred between *RB1*, D13S284 and D13S317.

The putative paternal haplotypes were [C-C-T]-213-11 and [C-C-T]-215-12, and the likely maternal haplotypes were [C-T-A]-213-9 and [C-C-T]-205-9. The mother could be a 9/9 homozygous for D13S317 or, alternatively, [C-C-T]-205-9 was a recombinant haplotype, in view that 213-9 cosegregation was more frequent than 205-9.

### Parental origin of mutations

The father was found to be a C/C homozygote at g.2056, while genotypic data indicated that the mother was also a C/C homozygote. Thus, g.2056C>G was not constitutional in the proband's progenitors and could have occurred either in the maternal or the paternal *RB1 *allele, as a *de novo *event in germ lines or in the zygote.

Alternatively, haplotypic data indicated that g.2056C>G could have occurred in the maternal *RB1 *allele because the expected paternal haplotype was [C-C-T]-213-11 and the expected maternal haplotype was [C-T-A]-213-9. A putative paternal haplotype, identical with the expected one, could be deduced in the proband, together with a putative maternal [G-T-A]-213-9 haplotype derived from the expected maternal haplotype.

Genotypic analysis indicated that both g.39606T and g.174351A alleles were of maternal origin because they were not present in the father. They were present in three quadruplets derived from two different embryos (A, C and D). Moreover, in the proband, in whom the paternal haplotype [C-C-T]-213-11 was deduced, these two mutations must have co-segregated with the maternal 213-9 alleles as was the case of quadruplets C and D. Moreover, the fact that child B did not carry these alleles ruled out the possibility of co-segregation with the paternal alleles 215-12 in B, C and D (Table 2).

### Association of mutations with retinoblastoma

We identified a novel constitutive mutation in the 5' UTR of the *RB1 *promoter region (g.2056C>G), in position – 4 with respect to the initial translation ATG codon in an infant, born after IVF, and affected with bilateral retinoblastoma. Mutations that weaken adherence to the Kozak consensus sequence in the 5'UTR have been found to negatively affect translation initiation. In eukaryotes, sequences flanking the AUG codon modulate their ability to halt scanning of the 40S ribosomal subunit and to initiate translation [14]. Translational efficiency was not herein analyzed, but Cs in these positions are highly conserved, being part of the consensus reference. This was confirmed by extensive analysis of Genbank data from 5'UTR sequences of some 700 vertebrate mRNAs [15]. Tumor samples were not available for analysis, a reason why studies of *RB1 *expression were not carried out with this material. cDNA analysis of blood RNA of the proband, however, indicated that only the g.2056C allele, in position -4 and located in the 5' UTR of *RB1*, was expressed, with an apparent null expression of the g.2056G allele.

Mutations detected in intron 3 (g.39606C>T) and intron 25 (g.174351T>A) in the proband and her two sisters (C and D) are less likely to be associated to retinoblastoma than g.2056C>G. The g.39606C>T mutation has been considered to be polymorphic [7] while g.174351T>A was also present as a polymorphic trait in unaffected individuals, with a frequency of 0.70 for the g.174351T allele and 0.30 for the g.174351A allele [12]. Conversely, g.174351T>A was associated to bilateral retinoblastoma in one patient in whom this mutation was constitutional and the only alteration to which retinoblastoma could be associated [11]. Interestingly, we also found this mutation as the only alteration in seven patients with retinoblastoma (Barbosa et al., unpublished data), a finding suggesting that g.174351T>A might be associated to development of retinoblastoma. Furthermore, the fact that g.174351T>A was also found in the unaffected quadruplets C and D does not exclude the possibility of retinoblastoma at older age.

### Genetic counseling and *in vitro *fertilization

In this study, retinoblastoma was detected in the proband at one month of age, a reason why the possibility of tumor development could not be excluded in her sisters. This is especially relevant for the monozygotic infants C and D who carried g.39606C>T and g.174351T>A and who must be followed by serial fundoscopic analyses. These mutations might be associated to retinoblastoma in view of previous reports and our unpublished observations.

With respect to IVF, the direct physical manipulation of gametes and embryos in the *ex vivo *environment might result in the appearance *de novo *point mutations resulting in the majority of genetic diseases in humans. Moreover, in a study of mouse embryos showed that genetic alterations in culture conditions might have considerable impact on the quality of embryos derived by ART [16].

## Conclusion

These results have implications for genetic counseling. Recurrent transmissions or potential transmissions of Mendelian disorders through the same gamete donor have been reported in ART [17]-[18] and constitutive *RB1 *mutations may appear *de novo *or be transmitted by one progenitor [4]. For establishing an accurate molecular diagnosis of retinoblastoma, genotypic data are necessary, mainly for distinguishing between hereditary from sporadic retinoblastoma. In this case, in which blood samples from the oocyte donor was not available, hereditary retinoblastoma could not be proven.

In ART, occurrence or risk of occurrence of a genetic disorder in the family must be calculated. Medical, family and reproductive histories of female and male donors must be obtained, and appropriate genetic counseling should be offered and genetic testing implemented [4]. These requirements cannot be met if the anonymity of gamete donors were to be respected with all ethical, legal, and psychological implications, thus making impracticable a precise diagnosis for genetic counseling. Our finding requires further research to confirm the association between IVF and retinoblastoma and to explore likely causal mechanisms.

## Competing interests

There are no competing interests (political, personal, religious, commercial, ideologic, academic or intellectual or any other) that may influence the authors' findings, opinions or points of view.

## Authors' contributions

RHB provided sequence and genotypic data of the individuals herein studied and analysed these findings. EL carried out fundoscopies and referred the patient and her family to FRV for Genetic Counseling. CRB acted as academic supervisor of RHB and contributed to the planning of this study. HNS was involved in drafting and revising the manuscript as well as giving final approval to the version to be published.

## Pre-publication history

The pre-publication history for this paper can be accessed here:


